# Intrahepatic cholangiocarcinoma detected on ^18^F-PSMA-1007 PET/MR imaging in a prostate cancer patient: a case report and literature review

**DOI:** 10.3389/fonc.2024.1408453

**Published:** 2024-06-12

**Authors:** Yu Sun, Haiyan Wang, Yihong Yang, Zhiwen You, Jun Zhao

**Affiliations:** Department of Nuclear Medicine, Shanghai East Hospital, Tongji University School of Medicine, Shanghai, China

**Keywords:** prostate cancer, prostate-specific membrane antigen, intrahepatic cholangiocarcinoma, hepatocellular carcinoma, positron emission topography

## Abstract

Radionuclide probes-targeted prostate-specific membrane antigen (PSMA) is used in diagnosis and treatment of prostate cancer (PCa). Recent studies have shown that PSMA is expressed in the tumor neovascular endothelium, such as in malignant liver tumors. We report a case of PCa with incidental intrahepatic cholangiocarcinoma (ICC) detection using ^18^F-PSMA-1007 and ^18^F-fluorodeoxyglucose (FDG) positron emission topography (PET)/MRI.^18^F-PSMA-1007 PET/MRI of our patient with PCa showed that one liver lesion had high PSMA uptake. ^18^F-FDG PET/MRI revealed minimal FDG uptake in the liver lesion. Histopathological examination revealed that the liver lesion was moderately to poorly differentiated cholangiocarcinoma. Our studies, along with others, demonstrated that malignant liver tumors, such as ICC, hepatocellular carcinoma (HCC), and combined hepatocellular-cholangiocarcinoma (CHC), and benign lesions, such as benign liver hemangioma, focal nodular hyperplasia, focal inflammation and steatosis, vascular malformation, and fatty sparing, exhibited elevated PSMA uptake. Moreover, PSMA-PET was superior to FDG-PET in detecting ICC and HCC, indicating that PSMA-PET may be used as alternative staging and to identify patients for PSMA-targeted therapy.

## 1 Introduction

Prostate-specific membrane antigen (PSMA) is a 100kDa type II-transmembrane glycoprotein which is overexpressed in nearly all prostate cancer cells (1). PSMA has been validated as a diagnostic and therapeutic target in prostate cancer (PCa), and radionuclide probes-targeted PSMA like ^18^F-PSMA, ^68^Ga-PSMA and ^177^Lu-PSMA was used in diagnosis and treatment of PCa (2). While PSMA is predominantly recognized in PCa, it is also expressed in various other solid tumors, such as thyroid, breast, liver, lung cancer, and glioblastoma (3–6). Primary liver cancer has shown the most rapid rise in mortality in decades. Hepatocellular carcinoma (HCC) is the most common primary liver cancer, followed by intrahepatic cholangiocarcinoma (ICC). Chen et al. (7) analyzed PSMA expression in 446 formalin-fixed paraffin-embedded (FFPE) liver tumors (213 HCC, 203 ICC, and 30 liver cirrhosis) and found that PSMA was expressed in 86.8% of HCC, 79.3% of ICC, and only 6.6% of liver cirrhosis. Few PSMA-targeted PET imaging studies of HCC, ICC, and combined hepatocellular-cholangiocarcinoma (CHC) have been reported. To date, only two cases have reported PSMA-PET imaging of the ICC. Herein, we report a case of PCa with incidental ICC detection using ^18^F-PSMA and ^18^F-fluorodeoxyglucose (FDG) PET/MRI.

## 2 Case presentation

**Figure 1:**
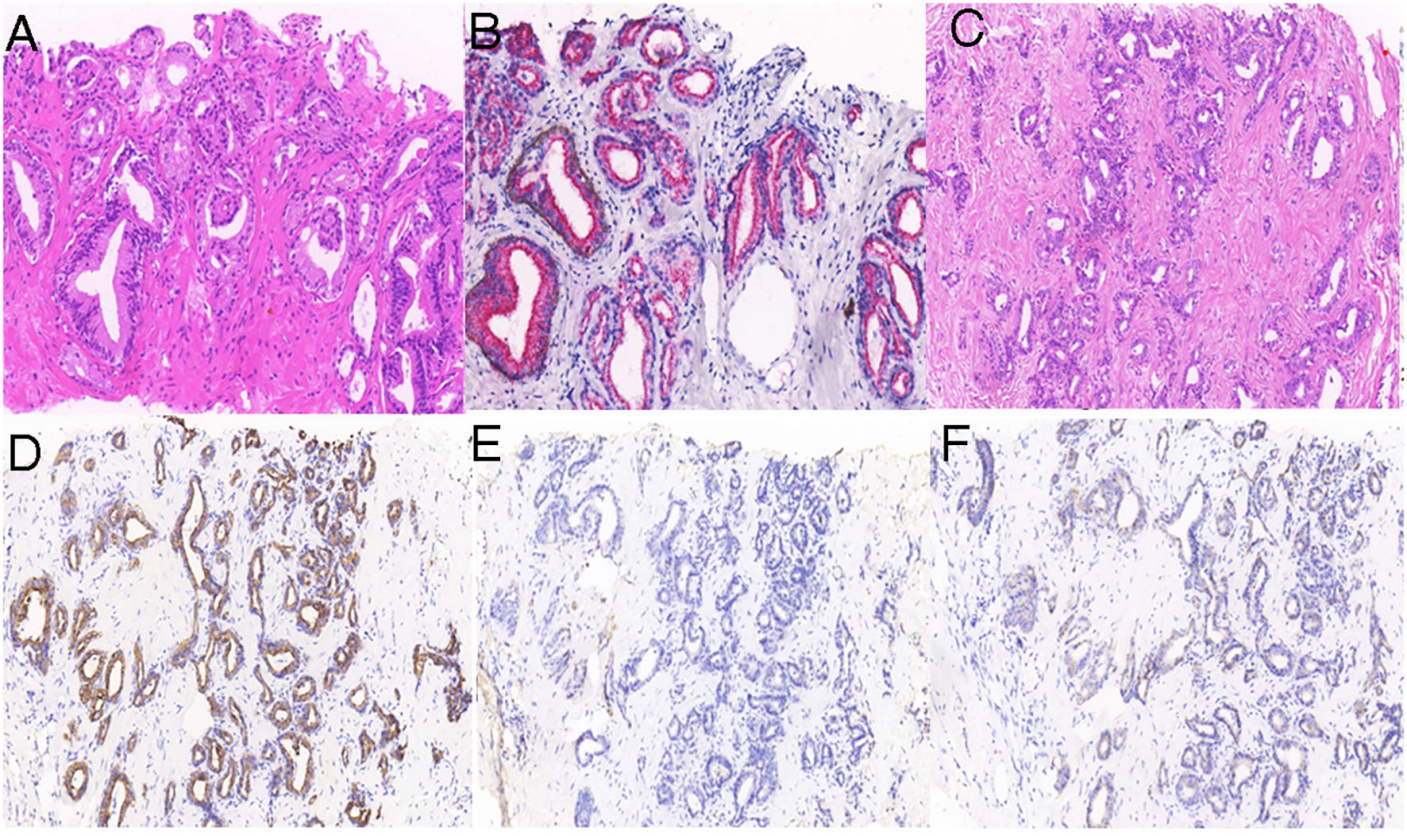
Histologic examination of the prostate and liver lesion. Hematoxylin and eosin (H&E) staining **(A)** revealed prostatic adenocarcinoma, with prostate tumor cells positive for P504S **(B)**. H&E staining **(C)** shows liver cholangiocarcinoma, with liver tumor cells positive for CK7 **(D)** and negative for PSA **(E)** and P504S **(F)**.

A 71-year-old man presented with more than 4 years history of urinary urgency and frequency, nocturia, and dysuria. The serum prostate-specific antigen (PSA) level was 5.54 ng/ml. Other tumor markers, including carbohydrate antigen (CA) 72–4 (75 U/ml), CA125 (37.3 U/ml), were elevated. Alpha-fetoprotein (AFP, 3.35 ng/ml), carcinoembryonic antigen (CEA, 2.98 ng/ml and CA19–9 (< 2.0 U/ml) levels were within normal ranges. Prostate biopsy was performed. Histopathological examination revealed prostatic adenocarcinoma with a Gleason score of 6 (3 + 3) (**Figure 1A**). Immunostaining revealed that the tumor cells were positive for P504S (**Figure 1B**). For preoperative staging, ^18^F-PSMA-1007 PET/MR imaging was performed.

**Figure 2:**
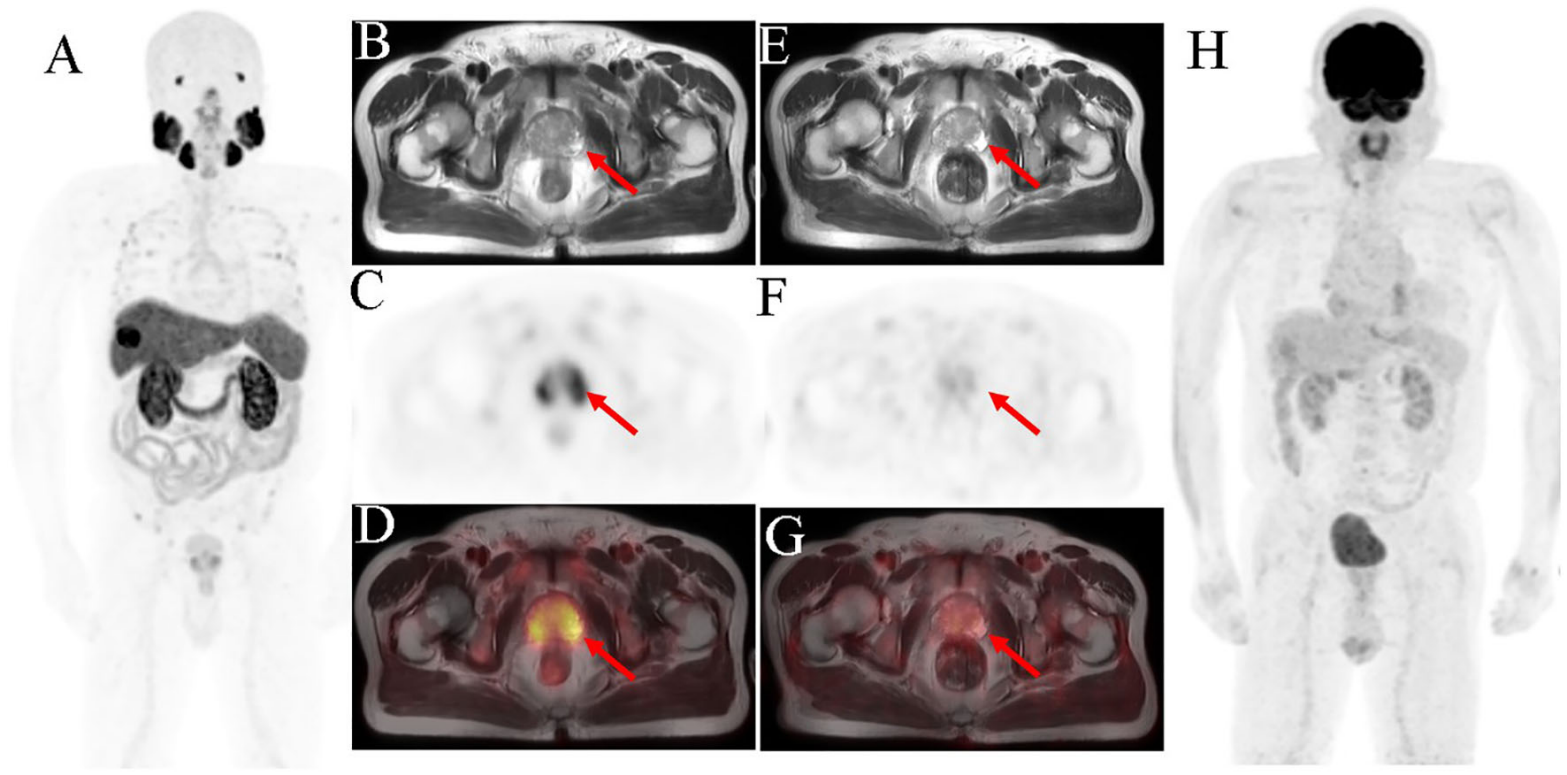
Prostate biopsy conducted in a 71-year-old man owing to high PSA level (5.54 ng/ml) and difficult, frequent, and urgent urination. The pathological result showed acinar adenocarcinoma, and the Gleason score was 6. ^18^F-PSMA-1007 PET/MRI imaging was performed for initial staging. The scan [**(A)** MIP image; **(B)** axial T2WI-MRI; **(C)** axial PET and **(D)** fused axial PET/MRI] shows high PSMA uptake in the bilateral peripheral zone of the prostate (SUVmax was 5.53). The ^18^F-FDG PET/MRI scan [**(E)** axial T2WI-MRI; **(F)** axial PET; **(G)** fused axial PET/MRI and **(H)** MIP image] shows no abnormal radiotracer concentration in prostate. The red arrow indicates the prostate.

**Figure 3:**
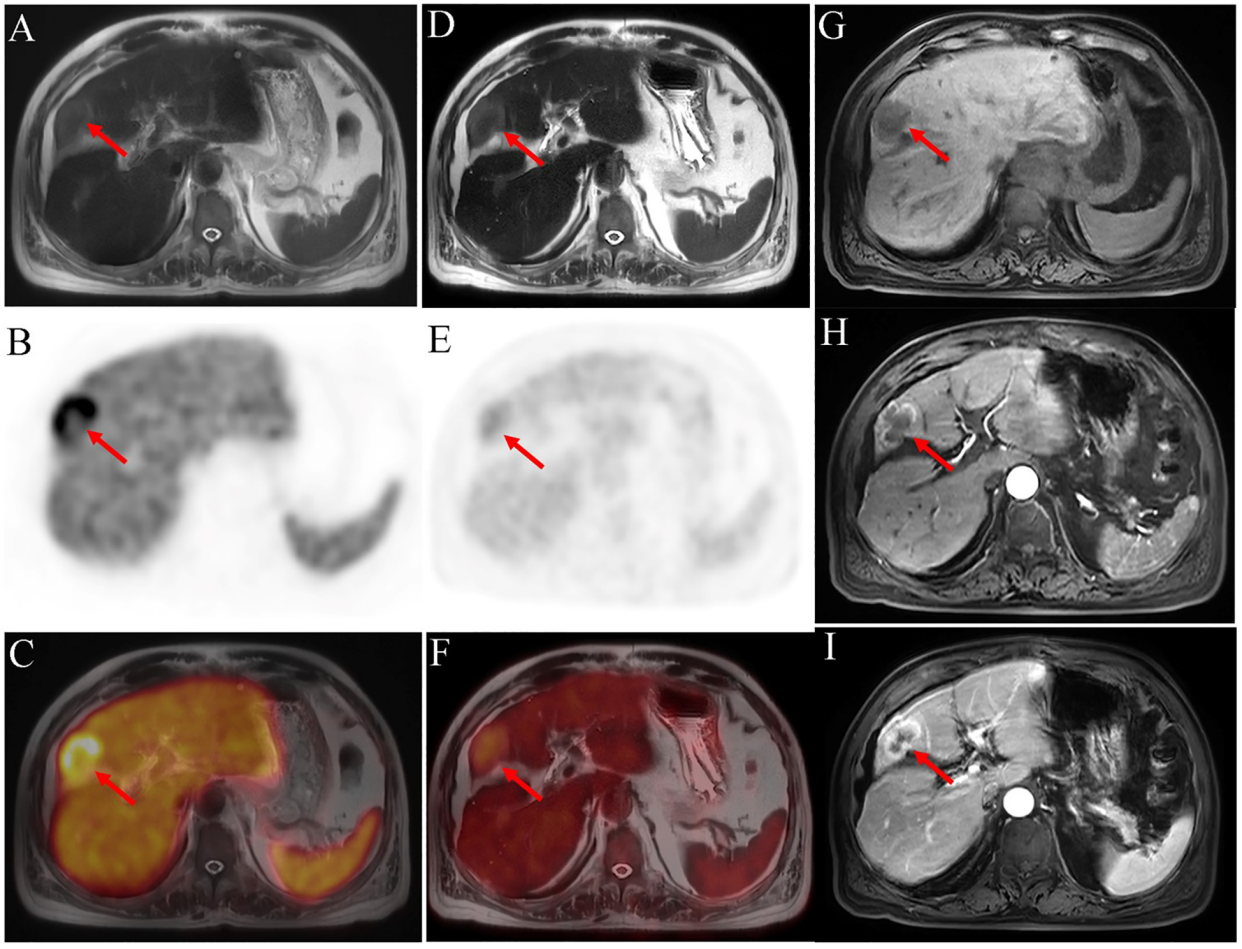
^18^F-PSMA-1007 PET/MRI [**(A)** axial T2WI-MRI; **(B)** axial PET and **(C)** fused axial PET/MRI] shows a high PSMA uptake mass in the liver (SUVmax=18.76). In ^18^F-FDG PET/MRI imaging [**(D)** axial T2WI-MRI; **(E)** axial PET and **(F)** fused axial PET/MRI], mild uptake is seen. Contrast-enhanced MRI of the abdomen [**(G)** T1WI-MRI; **(H)** arterial phase T1WI-MRI and **(I)** portal phase T1WI-MRI] reveals a significantly enhanced margin of mass in arterial phase, with further enhancement in the portal phase. The center of the mass is a necrotic area with no enhancement or radiotracer uptake. Postoperative pathology indicates a moderate to poor differential cholangiocarcinoma. The red arrow shows the liver lesion.

Whole-body PSMA-PET revealed diffuse increased uptake in the prostate (maximum standard uptake value [SUVmax]=5.53) (**Figures 2A, C, D**). T2-weighted MR revealed an area of low signal intensity in the bilateral peripheral zone and heterogeneous signal intensity in the transitional and central zones (**Figure 2B**). In addition to the prostate lesions, PSMA-PET imaging showed abnormally increased radiotracer uptake in the liver mass (SUVmax=18.76) (**Figures 3B, C**). MR imaging showed that the mass was located at the left-right lobe junction with a low signal on T1-weighted images (**Figure 3G**) and a slightly high signal on T2-weighted images (**Figure 3A**). To further determine the nature of liver lesions, ^18^F-FDG PET/MRI was performed. The prostate showed no FDG uptake (**Figures 2E–H**). The liver lesion had a low FDG uptake with an SUVmax of 3.23 (**Figures 3D–F**). Contrast-enhanced MRI of the abdomen was conducted revealing significant enhancement of the margin of the mass in the arterial phase, with further enhancement in the portal phase. The center of the mass was a necrotic area with no enhancement or radiotracer uptake (**Figures 3B, G–I**).

Subsequently, the liver lesion was surgically removed. Histopathological examination of the lesion revealed a moderately to poorly differentiated cholangiocarcinoma (**Figure 1C**); further, the tumor cells were positive for CK7 (**Figure 1D**) and negative for PSA (**Figure 1E**) and P504S (**Figure 1F**).

## 3 Discussion

PCa ranks first for estimated new cancer cases and second for estimated deaths for all cancer in men (8). Cancer metastasis is a major cause of mortality. Direct spread, hematogenous metastasis, and lymph node metastasis are metastatic pathways. PCa most frequently metastasizes to bones (84%), distant lymph nodes (10.6%), liver (10.2%), and thorax (9.1%). In patients with bone metastases, only 19.4% have multiple sites involved, and the most common sites of secondary metastases are the liver (39.1%), thorax (35.2%), distant lymph nodes (24.6%), and brain (12.4%) (9). In our patient, PSMA-PET showed multiple bones with PSMA uptake (data not shown) and the liver lesion with high PSMA and low FDG uptake. Therefore, we falsely concluded that the patient had bone and liver metastases. The misdiagnosis of this case led us to further search and summarize positive PSMA-PET images of liver lesions in PubMed. From 2015 to 2022, a total of 17 cases underwent PSMA-PET imaging (15 of ^68^Ga-PSMA and 2 of ^18^F-PSMA-1007), comprising eight cases of HCC (10–17), two of ICC (18, 19), one of combined hepatocellular-cholangiocarcinoma (CHC) (20), one liver metastasis of cholangiocarcinoma (CCA) (21), and five benign liver lesions (22–26), as detailed in **Table 1**. The mean age was 74.0 ± 6.4. The main purposes (76%, 13/17) of PSMA imaging were related to PCa, including elevated PSA levels and PCa staging or follow-up. The liver lesions in these cases were accidentally detected. Other reasons include research, staging, and therapy for malignant liver tumors. Most cases (n = 12) were concurrent with PCa. Two patients developed a third primary malignancy. Liver lesions were benign in five patients, including benign liver hemangioma (23), focal nodular hyperplasia (24), focal inflammation and steatosis (22), vascular malformation (25), and fatty sparing (26). All these 17 liver lesions had high PSMA uptake, and the mean SUVmax was 17.05 ± 6.31 (9.9–29.4). The median SUVmax of malignant tumor and benign lesion was 17.62 ± 7.62 and 16.1 ± 4.60, separately (P=0.76). Research had reported there was difference in SUVmax in ^68^Ga-PSMA and ^18^F-PSMA-1007 (27), so we just analyzed the SUVmax in ^68^Ga-PSMA. Five patients with malignant liver tumors underwent FDG examinations. The uptake in FDG-PET was lower than that in PSMA-PET in four patients. Three of them had HCC and one had ICC. Same as our case, Kang et al. reported incidental ICC in a 69-year-old man with PCa and the SUVmax of the liver lesion was 12.8 and 6.7 in ^68^Ga-PSMA and ^18^F-PSMA-1007 respectively (19). Another case also reported high PSMA uptake in ICC (18). Therefore, PSMA-PET imaging may be better than FDG-PET imaging for diagnosing and alternative staging of ICC. Until now, there was just three cases reported ICC in PSMA-PET, so that large sample should be further studied in the future.

**Table 1:** Reported cases of ^68^Ga/^18^F-PSMA scan in liver lesions.

	Investigation	Years	Age	Initial symptoms	PET scan purposes	Radioactive probe	Primary tumor	Liver lesions			PCa SUVmax	AFP
								Histopathology	PSMA SUVmax	FDG SUVmax		
HCC	Arun et al.	2015	78	anorexia, significant weight loss, hepatomegaly and obstructive urinary symptoms	elevated PSA (17ng/mL)	^68^Ga-PSMA	HCC	HCC	high	N.A.	N.A.	N.A.
	Sangeeta et al.	2016	77	N.A.	elevated PSA (40ng/mL)	^68^Ga-PSMA	PCa and HCC	HCC	15.7	N.A.	9.6	N.A.
	Hian et at.	2018	66	N.A.	follow-up of PCa	^68^Ga-PSMA	PCa and HCC	Well-differentiated HCC	high	N.A.	N.A.	N.A.
	Paola et al.	2019	87	N.A.	research purposes	^68^Ga-PSMA	melanoma and HCC	HCC	29.4	4.9	N.A.	N.A.
	Friedrich et al.	2020	69	N.A.	PCa staging	^68^Ga-PSMA	PCa, HCC and esophageal adenocarcinoma	HCC	high	slight	N.A.	N.A.
	Seval et al.	2020	74	right side pain, weight loss, and pruritus	alternative staging and therapy	^68^Ga-PSMA	HCC	HCC	20.3	7.6	N.A.	>20,000
	Zhao et at.	2020	77	bone pain	suspicion of PCa with bone metastasis, elevated PSA (53.32ng/mL)	^18^F-PSMA-1007	PCa and HCC	HCC	27.5	N.A.	11.7	108.2
	Sharjeel et al.	2021	82	N.A.	HCC restaging, elevated AFP (5752IU/mL)	^68^Ga-PSMA	HCC	HCC	high	N.A.	N.A.	5752
ICC	Rita et al.	2020	79	N.A.	follow-up of PCa, rising PSA	^68^Ga-PSMA	PCa, ICC and non-Hodgkin lymphoma	ICC	high	N.A.	N.A.	N.A.
	Kang et al.	2022	69	N.A.	PCa staging, elevated PSA (8.2ng/mL)	^68^Ga-PSMA	PCa and ICC	ICC	12.8	6.7	N.A.	N.A.
CHC	Ramin et al.	2017	70	N.A.	PCa staging, elevated PSA (8.1ng/mL)	^68^Ga-PSMA	PCa and CHC	CHC	9.9	N.A.	N.A.	N.A.
metastasis of CCA	Fahad et al.	2019	75	N.A.	therapy	^68^Ga-PSMA	CCA	metastasis	high	high	N.A.	N.A.
Benign	Hemant et al.	2016	77	N.A.	PCa staging	^68^Ga-PSMA	PCa	benign liver hemangioma	20.6	N.A.	N.A.	N.A.
	Seckin et al.	2020	66	N.A.	PCa restaging, elevated PSA (8.0ng/mL)	^68^Ga-PSMA	PCa	focal nodular hyperplasia	high	N.A.	N.A.	N.A.
	David et al.	2020	68	N.A.	follow-up of PCa	^18^F-PSMA-1007	PCa	hepatic focal inflammation, and steatosis	16.6	N.A.	N.A.	N.A.
	Sebastian et al.	2020	80	N.A.	follow-up of PCa, rising PSA (from 4.7-13ng/mL)	^68^Ga-PSMA	PCa	hepatic vascular malformation	16.3	N.A.	N.A.	N.A.
	Ramesh et al.	2022	64	N.A.	PCa staging	^68^Ga-PSMA	PCa	fatty sparing	11.4	N.A.	4.3	N.A.

PSMA, prostate-specific membrane antigen; PCa, prostate cancer; HCC, hepatocellular carcinoma; ICC, intrahepatic cholangiocarcinoma; CHC, combined hepatocellular-cholangiocarcinoma; CCA, cholangiocarcinoma; PSA, prostate-specific antigen; AFP, alpha-fetoprotein; SUVmax, maximum standardized uptake value; N.A., not available.

Three large-sample studies analyzed two different radiotracer PET imaging methods for detecting HCC. Kusymptcu et al. (28) found that FDG was positive in 15 patients and PSMA was positive in 16 patients. The mean SUVmax and tumor-to-background ratio of liver lesions on PSMA-PET were higher than those on FDG-PET. In nine patients, PSMA uptake was higher on visual and quantitative evaluations, whereas FDG uptake was observed in only four patients. A prospective pilot study analyzed 37 suspected malignant lesions in 7 patients with HCC and found that 36 of them showed increased PSMA uptake and only 10 were FDG-avid. A study with 14 HCC patients demonstrated that 36% of them had low FDG uptake, and 21% and 43% had moderate and high FDG uptake, respectively. On PSMA-PET, only 7% and 14% showed low and moderate uptake respectively, and 79% showed high uptake. The mean SUVmax and tumor-to-abdominal aorta or tumor-to-gluteal muscle ratios on PSMA-PET were higher than those observed on FDG-PET. These three studies showed that PSMA-PET imaging was superior to FDG-PET for detecting HCC. Its sensitivity, specificity, positive predictive value, negative predictive value, and accuracy are 97%, 100%, 100%, 80%, and 97%, respectively (10). Therefore, PSMA-PET may be used for the initial staging of HCC and as a potential 177Lu-PSMA therapy. Other than ICC and HCC, the CHC and liver metastasis of CCA also had high PSMA uptake (20, 21). Chen et al. (7) found that PSMA is primarily expressed in the neovascular endothelium associated with tumors. In benign liver lesions, this expression is probably due to increased local blood flow, local vascular permeability, and PSMA-expressing folate receptors in macrophages (29–31). Our study, along with others, revealed PSMA uptake in non-prostatic tissues and lesions, such as physiological uptake, benign pathological uptake, and non-prostatic uptake. Accordingly, when PET shows abnormal PSMA uptake in non-prostatic lesions in patients with prostate cancer, benign or malignant lesions other than metastases should be considered. A biopsy or surgery can be performed. If the non-prostatic lesion is located in the liver, it may be HCC, ICC, CHC, or other benign lesions. Although PSMA-PET has no advantage in the differential diagnosis of metastases of PCa and other lesions, it may be used as alternative staging and to identify patients with liver primary malignant for PSMA-targeted therapy.

## 4 Conclusion

Liver lesions with PSMA-avid in PCa cancer may not be metastasis of PCa but benign or malignant liver tumors, which should be further identified through pathology of biopsy or surgical specimens. PSMA-PET is superior to FDG-PET in detecting ICC and HCC and may be used as an alternative staging method.

## Data availability statement

The original contributions presented in the study are included in the article/supplementary material. Further inquiries can be directed to the corresponding author.

## Ethics statement

The studies involving humans were approved by Medical Ethics Committee of Shanghai East Hospital. The studies were conducted in accordance with the local legislation and institutional requirements. Written informed consent for participation in this study was provided by the participants’ legal guardians/next of kin. Written informed consent was obtained from the individual(s) for the publication of any potentially identifiable images or data included in this article.

## Author contributions

YS: Funding acquisition, Writing – original draft. HW: Writing – original draft. YY: Writing – original draft. ZY: Writing – original draft. JZ: Writing – review & editing.
